# ALDH1A2 (RALDH2) genetic variation in human congenital heart disease

**DOI:** 10.1186/1471-2350-10-113

**Published:** 2009-11-03

**Authors:** Marilene Pavan, Viviane F Ruiz, Fábio A Silva, Tiago J Sobreira, Roberta M Cravo, Michelle Vasconcelos, Lívia P Marques, Sonia MF Mesquita, José E Krieger, Antônio AB Lopes, Paulo S Oliveira, Alexandre C Pereira, José Xavier-Neto

**Affiliations:** 1 Laboratório de Genética e Cardiologia Molecular, InCor - HC FMUSP, São Paulo, Brasil; 2 Departamento de Biologia Celular e do Desenvolvimento, ICB-USP, Sao Paulo, Brazil; 3 Unidade Clínica de Cardiologia Pediátrica e Cardiopatias Congênitas, InCor HC-FMUSP, São Paulo, Brazil

## Abstract

**Background:**

Signaling by the vitamin A-derived morphogen retinoic acid (RA) is required at multiple steps of cardiac development. Since conversion of retinaldehyde to RA by retinaldehyde dehydrogenase type II (ALDH1A2, a.k.a RALDH2) is critical for cardiac development, we screened patients with congenital heart disease (CHDs) for genetic variation at the ALDH1A2 locus.

**Methods:**

One-hundred and thirty-three CHD patients were screened for genetic variation at the ALDH1A2 locus through bi-directional sequencing. In addition, six SNPs (rs2704188, rs1441815, rs3784259, rs1530293, rs1899430) at the same locus were studied using a TDT-based association approach in 101 CHD trios. Observed mutations were modeled through molecular mechanics (MM) simulations using the AMBER 9 package, Sander and Pmemd programs. Sequence conservation of observed mutations was evaluated through phylogenetic tree construction from ungapped alignments containing ALDH8 s, ALDH1Ls, ALDH1 s and ALDH2 s. Trees were generated by the Neighbor Joining method. Variations potentially affecting splicing mechanisms were cloned and functional assays were designed to test splicing alterations using the pSPL3 splicing assay.

**Results:**

We describe in Tetralogy of Fallot (TOF) the mutations Ala151Ser and Ile157Thr that change non-polar to polar residues at exon 4. Exon 4 encodes part of the highly-conserved tetramerization domain, a structural motif required for ALDH oligomerization. Molecular mechanics simulation studies of the two mutations indicate that they hinder tetramerization. We determined that the SNP rs16939660, previously associated with spina bifida and observed in patients with TOF, does not affect splicing. Moreover, association studies performed with classical models and with the transmission disequilibrium test (TDT) design using single marker genotype, or haplotype information do not show differences between cases and controls.

**Conclusion:**

In summary, our screen indicates that ALDH1A2 genetic variation is present in TOF patients, suggesting a possible causal role for this gene in rare cases of human CHD, but does not support the hypothesis that variation at the ALDH1A2 locus is a significant modifier of the risk for CHD in humans.

## Background

Congenital heart disease (CHD) was thought to be a multi-factorial, polygenic disease whose genes were as inscrutable as its postulated environmental agents [1]. Nowadays, some familial forms of CHD such as Tetralogy of Fallot (TOF), Atrial Septal Defects (ASD), Ventricular Septal Defects (VSD) and hypertrophic cardiomyopathy [2] are explained by reduced dosage (haploinsufficiency) of genes with major roles in cardiac development such as transcription factors [3,4], sarcomeric proteins [5], extracellular matrix proteins [6] and ionic channels [7].

Haploinsufficiency of a number of these genes can produce similar phenotypes (locus heterogeneity) and the same mutation is often not manifested (incomplete penetrance), or is expressed as a different anatomical/functional entity in the context of single families (variable expression) [8-10]. Therefore, CHD is heterogeneous even in the familial setting, suggesting that it has a more complex genetic basis than pure haploinsufficiency. It is likely that the instability caused by insufficient dosage of genes that are critical or permissive throughout cardiac development weakens cardiac morphogenesis, predisposing the embryo to a host of morphogenetic defects [4]. In this probabilistic view, defects could be triggered by unfavorable combinations of allelic variants of minor effect of cardiac development genes, by environmental insults such as changes in nutritional status (e.g. vitamin A) [11], or by exposure to toxic chemicals (e.g. alcohol) [12]. In this sense, the morphological consequences of these alterations would be contingent on the particular developmental processes that were at play when regulatory mechanisms were overcome.

Genes belonging to retinoic acid (RA) metabolic and signaling pathways are targets for investigation as direct causal agents, or modifiers of CHD. RA is a powerful morphogen utilized during early development for axial patterning and, at later stages, for organogenesis. The vertebrate heart is particularly affected by variations in RA signaling [13] and either deprivation, or excess of its precursor (Vitamin A or retinol) at multiple developmental stages produces cardiac and vascular malformation in animal models [14].

RA is a carotenoid that cannot be synthesized *de novo *by animals and thus, must be obtained from preformed animal-derived precursors such as retinyl esters and retinol (Vitamin A) [15], suggesting a direct link between nutritional status and teratogenesis. There are multiple metabolic routes to generate RA and these pathways require oxidative steps (reviewed in [16]). In humans, RA is synthesized from vitamin A (retinol). Retinol freely crosses membranes, but it is also transported to cells by retinol binding protein (RBP), which transfers retinol to cells via STRA-6, a membrane receptor [17]. STRA-6 is a target in congenital human diseases manifested by defects in eye, lung, heart and skeletal muscle, as well as by mental retardation [18,19]. Within cells, retinol is successively oxidized to retinaldehyde and, then, to RA. The first step is reversible and is catalyzed by alcohol dehydrogenases (ADHs), or by short-chain dehydrogenases/reductases (SDRs) [20]. The second step is the irreversible conversion of retinaldehyde to RA. In vertebrates this reaction is catalyzed by retinaldehyde dehydrogenases (a.k.a. RALDHs) that belong to two aldehyde dehydrogenase (ALDH) families, the ALDH1 s (all-trans and 9-cis retinaldehyde dehydrogenases ALDH1A1, ALDH1A2 and ALDH1A3) and the 9-cis retinaldehyde dehydrogenase ALDH8 s (a.k.a. RALDH4) [16]. Among RALDHs, ALDH1A2 is the major form involved in early embryonic and in cardiac development [21,13]. The most severe form of RA signaling deprivation is the genetic deficiency of ALDH1A2, which is lethal at 8.5 days post-fertilization in mice [13,22].

In this study we used ALDH1A2 as an entry point to understand the relationship between RA metabolism and human CHD. Our goal was to establish whether the ALDH1A2 gene is a target for genetic alterations that could produce or, at least, modify the expression of human CHD. We utilized a two-pronged strategy based on a genetic screen to detect mutations carrying the potential to alter enzyme activity and a family-based genetic association study to inquire whether genetic variation at the ALDH1A2 locus modulates the risk to develop CHD.

## Methods

### Patients

A total of 133 trios (non-syndromic congenital heart disease patients with their parents) and seen at the Pediatric Cardiology Outpatient Clinic of the Heart Institute, São Paulo, Brazil were invited to participate in the study. Venous blood was obtained for genomic DNA extraction from each participant of the study. The protocol was approved by the Institutional Review Board of the University of São Paulo Medical School and all participants read and signed an approved informed consent.

### PCR and DNA sequencing

Genomic DNA was extracted from peripheral blood leukocytes by means of a salting-out procedure [23]. We have created specific genotyping assays for each genotyped marker. Briefly, the studied polymorphisms were detected by polymerase chain reaction-restriction fragment length polymorphism assay (PCR-RFLP). A 30-cycle PCR was carried out in a PTC-DNA Engine Tetrad2 using a 10 μL reactive solution containing 10 mm Tris-HCl (pH 9.0), 50 mm KCl, 2.5 mM MgCl2, 100 μM of each dNTP, 0.3 U Easy Taq DNA Polymerase, 5 pmol of each primer and 1 μL of genomic DNA template. PCR products were digested with 1 U of restriction enzyme and visualized by 3% agarose gel electrophoresis. Quality control for these assays was assessed by randomly selecting and re-assaying 40 samples. To genotype the A151G polymorphism we employed a direct sequencing strategy using primers directed against flanking regions of human ALDH1A2 exon 4. The DNA sequences were determined by bi-directional sequencing. For sequencing, the PCR products were purified with E.Z.N.A. Cycle-Pure Kit (200), Omega Bio-Tek, USA, according to manufacturer's instructions. Bidirectional direct sequencing was carried out using the Applied Biosystems (ABI, Foster City, CA) v3.1 Dye-Terminator Sequencing Kit and an ABI 377 Sequencer. The same strategy was employed to sequence the ALDH1A2 gene (see additional file 1 - Supplemental table S1).

### Bioinformatics tools

The localization (chromosome 15) and structure of ALDH1A2 gene was obtained from Ensembl http://www.ensembl.org/Homo_sapiens/geneview?gene=ENSG00000128918. For describing DNA changes we have used the HGVS nomenclature http://www.hgvs.org. NCBI reference sequences used were NM_003888.2 and NP_003879.2. For our family-based study we chose a set of polymorphic markers that spread throughout the ALDH1A2 gene. These markers were chosen from the dbSNP database http://www.ncbi.nlm.nih.gov/SNP and included only those with suitable frequencies for genotyping. Primer pairs were designed with the Interactive software Primer Design - GeneFisher http://www.genefisher.de. For the splicing site analysis we used the ESEfinder program, available at http://rulai.cshl.edu/cgi-bin/tools/ESE3/esefinder.cgi?process=home. To evaluate the structural consequences of the sequence alterations we determined on RNA folding we used the Genebee program http://www.genebee.msu.su/. Linkage disequilibrium structure determination was conducted using the Haploview 3.2 software.

### Association study design

To determine the linkage disequilibrium structure of the ALDH1A2 locus in our population we selected 6 SNPs that cover the genomic region harboring the human ALDH1A2 gene (see additional file 1 - Supplemental table S2), including the genetic marker rs16939660, previously associated to spina bifida [24]. The linkage disequilibrium information was used to optimize our tests for the possible association between genetic variability at the ALDH1A2 locus and CHD through a transmission disequilibrium test (TDT).

### pSPL3 splicing assay

Fragments of the human ALDH1A2 gene containing the wild type exon 4, p.Ala151Ser or p.Ala151Ala silent mutant, flanked by about 450 bp of intronic sequences were cloned into the splicing vector pSPL3 [25], generating plasmids WT-ALDH1A2 Exon 4-pSPL3 and MTs-ALDH1A2 Exon 4-pSPL3. In pSPL3, two short exons of the HIV *tat *gene are separated by a long intron, which is excised upon splicing to give rise to mature mRNA containing exon 1 and 2. Genomic fragments inserted into the pSPL3 intron are spliced according to the potency of their splicing signals, providing an assay for mutations that affect splicing efficiency [25]. Plasmids were transfected into HEK 293 human embryonic cells using calcium phosphate precipitation [26]. After 48 hours mRNA was extracted from cells and reversed-transcribed to generate templates for PCR amplification with primers flanking pSPL3 exons 1 and 2. PCR conditions were adjusted to produce amplicons in the linear range. PCR reactions were performed at 35 cycles (n = 5). Quantification was performed in ethidium bromide-labeled agarose gels with the Scion Image program (ported from NIH Image for the Macintosh by Scion Corporation). Results are represented as the percentage of exon 4 exclusion in pSPL3-derived amplicons. Data are expressed as mean ± standard error of the mean (SEM).

### ALDH sequences and phylogeny

*Homo sapiens*, *Mus musculus*, *Gallus gallus*, *Xenopus tropicalis *and *Danio rerio *genomes were examined for ALDH sequences and phylogenetic trees were constructed from an ungapped alignment containing ALDH8 s, ALDH1Ls, ALDH1 s and ALDH2 s. Trees were generated by the Neighbor Joining method (NJ) [27] and were rooted with an outgroup formed by ALDH8 s. Nodes with bootstrap inferior to 80% were collapsed.

### Cloning, cell culture and mRNA extraction

The ALDH1A2 gene exon 4 was PCR amplified from genomic DNA from a heterozygous patient for the silent p.Ala151Ala mutation. The amplified fragments were then cloned into the PCR-Script plasmid (PCR-Script Amp Cloning Kit - Stratagene, CAT# 21189.5). Cloned inserts were sequenced to certify that mutations were not generated during the PCR amplification step. The fragments were then subcloned into *Eco*RV and *Sst*I sites from the splicing vector pSPL3 [25].

HEK 293 cells were cultured in DMEM medium supplemented with 10% of FBS at 37°C, in 5% of CO^2^. Six-well plates were utilized and the cells were cultured up to 75% of confluence. For transfection, we utilized Lipofectamine (Invitrogen™/cat. 11668-019). Each well was transfected with 1,6 ug of plasmid DNA.

RNA was extracted 48 h after transfection with Trizol reagent (GIBCO BRL, USA) according to the manufacturer's instructions. The following sequences of primers were utilized to amplify the cDNA fragments. SA2: ATC TCA GTG GTA TTT GTG AGC; SD2: GTG AAC TGC ACT GTG ACA AGC. Amplified PCR products were visualized by 1% agarose gel electrophoresis and stored in digital format.

### Structural analyses

To evaluate how mutations Ala151Ser and Ile157Thr may impact protein folding, we built two structural models for ALDH1A2mutant proteins. For that we employed homology modeling based on the published structure of *Rattus norvegicus *ALDH1A2 (PDB id 1BI9) as a template, using the Nest program from Jackal package [28]. The stereo chemical quality of the structures was analyzed with Procheck [29]. The leap program, of AMBER 9 package [30] was used to prepare the structure for molecular mechanics (MM) simulations. MM was carried in explicit solvent and MM trajectories were calculated using Sander and Pmemd programs, of AMBER 9 package. A cutoff of 8 Å was used for electrostatic interactions. The solvated complex was equilibrated by carrying out a short steepest descent minimization, followed by 50 ps of heating (0 to 300 K) and 50 ps of density equilibration with weak restraints on the complex, ending with 6 ns of conformational exploration at 300 K. All simulations were run using the shake approach on hydrogen atoms, a 2 fs time step, and Langevin dynamics for temperature control. We calculated the potential energy for 600 monomer conformations wild type and mutant structures produced along the MM using the MM_PBSA program of AMBER9 package. To find the more energetically stable regions in the conformational space we estimated the inverse coefficient of variation (CV^-1^) for each 1 ns window of the MM simulation run. The most stable monomer configurations were identified as those with the lowest mean energy and highest CV^-1 ^Mutant dimer and tetramer models were built using non-crystallographic symmetry obtained from PDB 2BI9 for monomer configurations of lowest energy. Dimer and tetramer models were analyzed for structural hindrances.

### Statistical analysis

Allele and genotype frequencies were calculated through gene counting. Hardy-Weinberg equilibrium was tested with the Chi-square test. We used Haploview 3.2 software for linkage disequilibrium and haplotype determination. A possible association between presence of a particular genotyped allele and disease was tested through the TDT test, performed with Haploview 3.2. Haplotype analyses were conducted using the Haploview TDT association test. We considered p values less than 0, 05 on a 2-sided test as significant. Using the Quanto software Version 1.2.3 and considering the tested sample and markers we anticipate an eighty percent statistical power to detect an effect size (odds ratio) of 3,0 for the rarer observed haplotype (0,05) and 2,3 for the commonest observed haplotype (0,31).

## Results

### Mutation screening in CHD

To test whether the ALDH1A2 gene is a target for mutations in human CHD we employed DHPLC (denaturing high performance liquid chromatography) [31] followed by bi-directional DNA sequencing in a group of patients with multiple CHD etiologies (see additional file 1 - Supplemental table S3). DNA fragments corresponding to all ALDH1A2 exons were PCR amplified and submitted to DHPLC, and those displaying altered elution profiles were selected for bi-directional sequencing. This first screening in a heterogeneous CHD setting revealed 6 alterations: one c.A453G transition previously characterized as a synonymous, p.Ala151Ala, polymorphism (rs16939660) associated with spina bifida [24] and five intronic alterations (Table 1). None of these changes occurred within the Kozak sequence. As these alterations are potentially associated with only a modest to low risk of genetic defects [32], we hypothesized that the failure to detect ALDH1A2 variants associated with high risk of CHD was due to insufficient numbers of patients in each of the CHD categories represented. Therefore, we next explored ALDH1A2 alterations in a group constituted only by TOF patients.

**Table 1:** Variations found in DHPLC screening.

SNP	CHD chromosomes	Non-CHD chromosomes
c.A453G (exon 4)*(rs16939660)*	1/166 (0,6%)	0/200 (0%)

rs35667670 intron 2	3/146 (2%)	3/92 (3,26%)

c.160-422G > A (intron 3)	80/158 (50,63%)	41/88 (46,6%)

c.25-422G > C (intron 3)	1/166 (0,6%)	2/100 (2%)

c.116+613G > T (intron 5)	63/156 (40,38%)	44/94 (46,80%)

c.92+742_93+742insA (intron 6)	1/166 (0,6%)	0/100 (0%)

rs3784259 (intron 8)	75/158 (47,48%)	46/92 (50%)

In this study we found 5 intronic alterations and one polymorphism (c.A453G) in ALDH1A2 exon 4.

### Mutation screening in Tetralogy of Fallot

**Figure 1:**
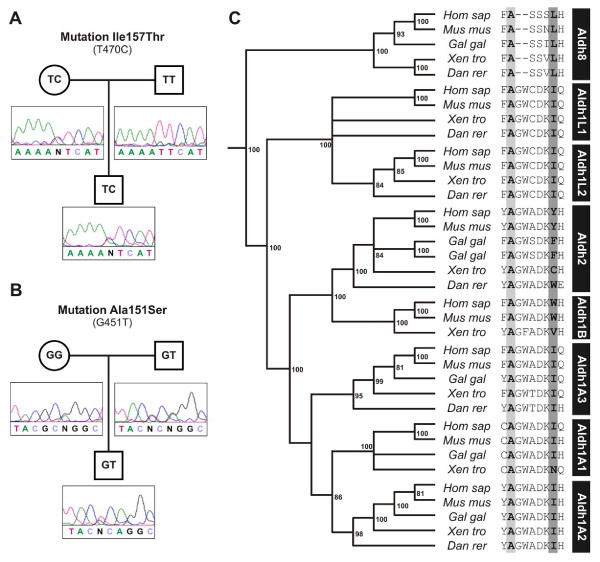
**Aldh1a2 mutations in Tetralogy of Fallot: evolutionary conservation of affected amino acids**. A) The T to C transition at nucleotide 470 changes an ATT cistron into ACT, leading to the substitution of an Isoleucine at position 157 by a Threonine. The p.Ile157Thr was traced back to a maternal allele. B: The G to T transversion at nucleotide 451 changes a GCA cistron into TCA, leading to the substitution of an Alanine at position 151 by a Serine. The p.Ala151Ser mutation was traced back to a paternal allele. Interestingly, one paternal allele and one maternal allele both display an A to G transition at nucleotide 453, producing the p.Ala151Ala silent mutation (rs16939660). C) An alignment representing vertebrate Aldh1a1-3 (all-trans and 9-cis retinaldehyde dehydrogenases), the closely related ALDH2 s and ALDH1B1 s, as well as Aldh1Ls (Tetrahydrofolate Dehydrogenases) and ALDH8 s (9-cis retinaldehyde dehydrogenases) as outgroups. The alignment indicates that Ala151 (dark shading) is highly conserved in all ALDHs represented, while Ile157 is conserved in all vertebrate ALDH1As (light shading). Numbers displayed in the phylogenetic tree represent bootstrap support for the nodes represented.

Fifty TOF patients, tested negative for chromosome 22q11.2 microdeletion [33], were examined by direct bi-directional sequencing of ALDH1A2 exons. This second screen identified two non-conservative mutations at exon 4, which encodes part of the tetramerization domain, an oligomerization surface responsible for assembly of enzyme dimers and tetramers [34]. A c.T470C transition (p.Ile157Thr) changed a non-polar isoleucine residue to a polar threonine and a c.G451T transversion changed a non-polar alanine to a polar serine (p.Ala151Ser) (Figures 1A and 1B). These alterations were not observed in 150 normal individuals (300 chromosomes) screened, nor in the first 83 individuals with diverse forms of CHD or in the remaining 48 TOF sequenced cases (summing up 262 chromosomes) and have not been described before in open-source SNP databases.

We also identified a conservative valine to isoleucine mutation at exon 9 (p.Val348Ile), which was previously characterized as a common variant rs4646626; an A to G transition 6 bp upstream from the transcription start, previously described as polymorphism rs34645259; and a synonymous SNP p.Ile418Ile at exon 11, previously characterized as polymorphism rs35251510.

The patient harboring the p.Ala151Ser substitution was a 21-year-old man, the first child of non-consanguineous parents. He was born at term and was diagnosed with TOF at the age of 2 months. His milestones were normal and he has normal intelligence. He was referred to our service at the age of 1 year and 10 months for surgical correction. On physical examination, his weight was 7.2 kg, height, 77 cm. He had no dysmorphic features. Laboratory tests included an echocardiogram that revealed subaortic VSD, severe pulmonary stenosis (systolic gradient of 85 mmHg) with a pulmonary artery truncus of 1.2 cm and pulmonary artery branches of 1.0 cm. There were no coronary anomalies and no aortopulmonary collateral arteries. Abdominal and renal ultrasounds were normal. The skeletal survey was unremarkable. The Ala151Ser mutation was also present in the genome of his father (Figure 1B).

The patient harboring the Ile157Thr substitution was an 18-year-old man, the second child of non-consanguineous parents. He was born at term with a birth weight of 3,300 g and was diagnosed with a heart murmur at the age of 15 days. His milestones were all normal and he has normal intelligence. He was referred to our service at 1 month for a congenital heart disease diagnosis workup. On physical examination, his weight was 3.870 kg, height, 55 cm. He had no dysmorphic features. No clinical stigmata of Noonan/Noonan-like syndrome or Beckwith-Wiedemann syndrome were present. Laboratory tests included an echocardiogram that revealed TOF. While preparing for surgical correction, abdominal and renal ultrasounds detected a kidney mass, eventually diagnosed as Wilms tumor. Wilms tumor was unilateral and was treated with chemotherapy and surgery at the age of 3 years. Skeletal surveys disclosed no abnormalities. Tetralogy of Fallot was corrected (total correction) at the age of 4 and, interestingly, agenesis of the left pericardium was observed during corrective surgery. The Ile157Thr mutation was also present in the genome of his mother.

### Evolutionary conservation of ALDH1A2 amino acids mutated in TOF

Non-conservative mutations are low frequency alterations that carry a moderate to high risk of genetic defects, depending on the location and function of the amino acid in the protein structure [32]. Very often, the structural and functional importance of a particular amino acid is underscored by its evolutionary conservation. To evaluate the extent to which Ala151 and Ile157 are conserved, and, therefore, more likely to be required for protein structure and function, we produced an alignment of vertebrate ALDH1As. This alignment also contains the closely related ALDH2 and ALDH1B1 genes, together with ALDH1Ls (formyl tetrahydrofolate dehydrogenases) and ALDH8 s (9-cis retinaldehyde dehydrogenases) as outgroups.

As indicated in Figure 1C Ala151 is conserved in all vertebrate ALDH1A2 s, as well as in its paralogs, ALDH1A1 s and ALDH1A3 s. Ala151 is also conserved in ALDH2 s, ALDH1B1 s, as well as in ALDH8 s and is the predominant residue in formyl tetrahydrofolate dehydrogenases (ALDH1L1s), which are important players in folate metabolism and possess an ALDH domain closely related to ALDH1 s (figure 1C).

Ile157 is conserved in all vertebrate ALDH1A2 s, as well as in ALDH1A3 s and is the predominant residue in ALDH1A1 s, except in frogs, which display a divergent asparagine (*X.tropicalis*), or a closely related valine (*X.laevis*) (Figure 1C and data not shown). Interestingly, this residue is the predominant amino acid in ALDH1L1 s, while in the closely related ALDH2 s and ALDH1B1 s the position is occupied by aromatic residues, or Histidine and Glutamine, respectively (Figure 1C).

In sum, p.Ala151Ser is a non-conservative mutation in an amino acid that has been deeply conserved in the evolution of the ALDH super family, while p.Ile157Thr has been conserved in vertebrates at least since the divergence of the sarcopterygean and actinopterygean lineages of bony fishes 450 millions of years ago [35].

### Structural roles of ALDH1A2 amino acids mutated in TOF

**Figure 2:**
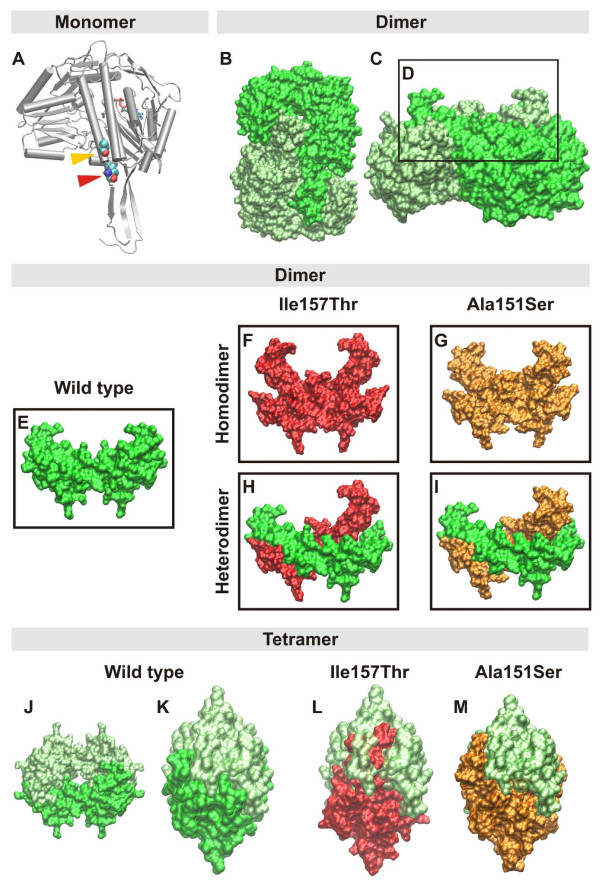
**Effects of p.Ala151Ser and p.Ile157Thr mutations in the predicted structure of human ALDH1A2**. A) Cartoon representation of the ALDH1A2 monomer, highlighting p.Ala151Ser (orange arrowhead) and p.Ile157Thr (red arrowhead) mutations. B-C) Surface mode views of wild type Aldh1A2 dimers. D) The boxed structure highlights homodimer surfaces that take part in the process of tetramerization. E-I) Hetero- and homodimer Aldh1A2 configurations produced in homozygote and heterozygote individuals for the p.Ala151Ser and p.Ile157Thr mutations. Wild type and mutant homo- and heterodimer tetramerization surfaces are represented as in D. The asterisk indicates the major conformational changes observed in the tetramerization domains of p.Ala151Ser (orange) and p.Ile157Thr (red) mutants in homo or heterodimer configurations. J) Homotetramer model of wild type human Aldh1A2. K) 90° rotated Y axis view of the wild type human Aldh1a2 homotetramer as shown in J. L) Ile157Thr-wild type heterotetramer showing large areas of stereo chemical hindrance. M) p.Ala151Ser-wild type heterotetramers showing minor departures from the wild type conformation.

Non-conservative mutations in gene coding regions potentially affect protein function, as they may alter protein structure. To evaluate how mutations p.Ala151Ser and p.Ile157Thr impact protein structure, we built two structural models for ALDH1A2mutant proteins.

p.Ala151Ser is located on alpha-helix-C, which contributes one of the sides of the catalytic channel pocket and also anchors the three-stranded beta-sheet that forms the oligomerization domain (orange arrowhead, Figure 2A). [34,36].

p.Ile157Thr occupies a position between the C-terminal capping of alpha-helix-C (Lys156) and the first beta-strand of the oligomerization domain (red arrowhead, Figure 2A) [34,36]. In the native protein, Ile157 forms a hydrophobic cluster with Leu500, Trp153 and Tyr150.

To explore whether subunit association may be affected by disruption of polar and/or hydrophobic interactions at the oligomerization surfaces, or close to them, we screened the conformational space of wild-type and mutant ALDH1A2 structures with molecular mechanics (MM) simulations. In these analyses the wild-type structure yielded four highly stable conformations (-8,264 Kcal/mol for the most stable of them) (Figure 2E), while p.Ala151Ser and p.Ile157Thr mutant proteins produced four and seven highly stable conformations, with -8,105 Kcal/mol and -8,296 Kcal/mol for the most stable conformations of p.Ala151Ser and p.Ile157Thr mutants, respectively (see additional file 2 and 3 - Supplemental Figure S1).

The superimposition of stable mutant structures upon the native structure generated RMSDs (root mean square deviations) of 3.18 Å and 7.36 Å for Ala151Ser and p.Ile157Thr, respectively, indicating that p.Ile157Thr produces more pronounced structural changes than p.Ala151Ser. Since both p.Ala151Ser and p.Ile157Thr are located at the tetramerization domain, which is the oligomerization structure responsible for formation of protein dimers and tetramers, we evaluated whether these mutations would interfere with the ability of monomers to associate in dimers (Figure 2E-I), or with the capability of dimers to join in as tetramers (Figure 2J-M). We first evaluated the effects of mutations upon dimer formation. As indicated in Figure 2F and 2G, both mutations change the conformation of the three stranded beta-sheet of the tetramerization domain. This disrupts the delicate interaction network that occurs between the dimerization surfaces of interacting monomers (Figure 2H and 2I). By its turn, disruption of interactive surfaces induces alterations in dimer coupling that are also reflected in the protein surfaces utilized for tetramerization These effects are demonstrated in Figure 2L, which depicts a large clashing region that includes the three-stranded beta-sheets of the tetramerization domains of p.Ile157Thr mutant and wild type heterotetramers. In summary, the stereo chemical conflicts associated with the p.Ile157Thr mutation are much larger than those associated with the p.Ala151Ser mutation (Figure 2M).

### Splicing effects of Aldh1a2 mutations in TOF

**Figure 3:**
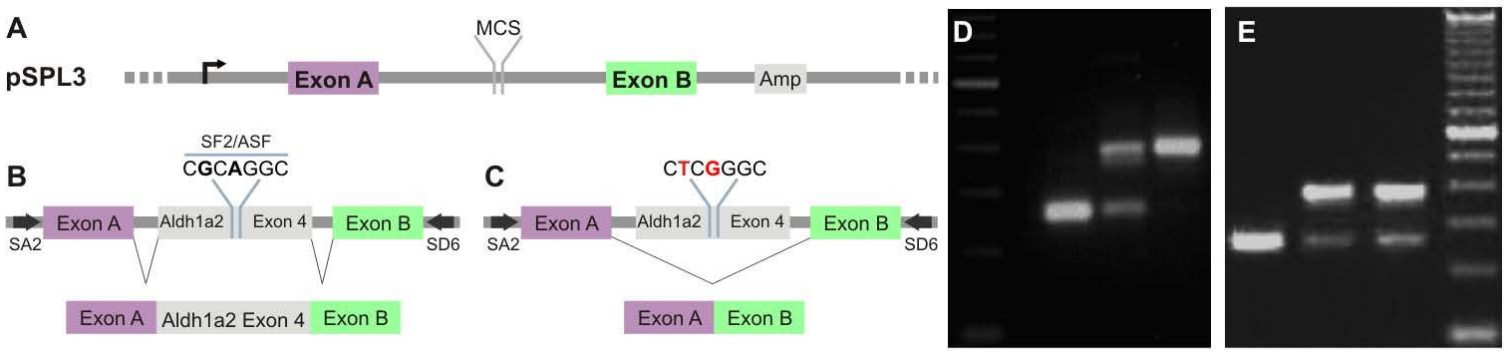
**Mutations at Aldh1A2 exon 4 splicing enhancer alter splicing efficiency**. A) c.G451T and c.A453G of the Aldh1a2 exon 4 interrupt a putative binding site for the splicing factor SF2/ASF. B) In pSPL3 splicing assay a long HIV *tat *intron is flanked by two exons containing weak splicing signals. Fragments inserted within the pSPL3 intron are spliced according to the potency of their signals. C) If splicing signals in wild type ALDH1A2 exon 4 are strong, the assay will produce a 250 bp PCR fragment containing Aldh1a2 exon 4 flanked by HIV exons 1 and 2. If exon 4 mutants reduce the efficiency of splicing signals a PCR fragment containing HIV exons 1 and 2 + ALDH1A2 exon 4 will be produced (380 bp). D) pSPL3 assays with HEK 293 cells indicate that the wild-type exon 4 contains weak splicing signals, as indicated by production of PCR fragments without exon 4 sequences. The c.G451T transversion strengthens exon 4 splicing signals, as indicated by the disappearance of the smaller (250 bp) fragment. First lane, 100 bp ladder; second lane, pSPL3 vector; third lane pSPL3 vector + fragment of the human ALDH1A2 gene containing the wild type exon 4 flanked by about 450 bp of intronic sequences; forth lane, pSPL3 vector + fragment of the human ALDH1A2 gene containing the c.G451T transversion, flanked by about 450 bp of intronic sequences. E) The c.A453G transition is associated with a small, non-significant decrease in splicing strength (4%). First lane, pSPL3 vector; second lane, pSPL3 vector + fragment of the human ALDH1A2 gene containing the wild type exon 4 flanked by about 450 bp of intronic sequences; third lane, pSPL3 vector + fragment of the human ALDH1A2 gene containing the c.A453G, flanked by about 450 bp of intronic sequences.

Mutations in nucleotidesthat are part of splicing signals are frequent in genetic diseases (Krawczek et al., 1992). To evaluate a possible splicing involvement of the ALDH1A2 alterations that we detected in TOF, we tested whether they would map to known binding sites for exonic splicing enhancers (ESEs), or for exonic splicing silencers (ESSs). This analysis indicated that the non-conservative p.Ala151Ser (G451T transversion) and the synonymous mutation p.Ala151Ala (c.A453G transition) both map to a putative binding site (CGAAGGC) for the splicing factor SF2/ASF within exon 4 (Figure 3A-C). Because of the link reported here between p.Ala151Ser and TOF, and of the reported association of p.Ala151Ala with spina bifida [24], we set out to investigate the effect of these mutations in exon 4 splicing.

Using the pSPL3 splicing assay [25] in human embryonic kidney 293 cells (HEK), we showed that the splicing signals of exon 4 are only of moderate strength, as demonstrated by exclusion rates of 20,9 ± 1,6%, range 13,5 to 35,9%). This finding is consistent with the description of an ALDH1A2 EST (DA747232), in which exon 4 is missing from the transcript [37]. As demonstrated in Figure 3D, the p.Ala151Ser (c.G451T transversion) completely rescues exon 4 splicing, increasing the proportion of the correctly spliced version from 76% to 100%. This indicates that the mutation increased, rather than decreased, the splicing strength of exon 4. In contrast, the p.Ala151Ala/c.A453G polymorphism is not associated with significant changes in the ability to correctly splice exon 4 (28,0 ± 2,9% exclusion of exon 4 for mutant c.A453G, versus 24,0 ± 2,8% for the wild type; p = 0.36; two-tailed, paired T-test) (Figure 3E).

Since synonymous changes can also affect splicing function by interfering with mRNA secondary structure [38], we investigated whether the c.A453G transition was consistent with significant changes in this parameter. The results of these investigations are described in Supplemental Figure S2 (see additional file 2 and 4 - Supplemental Figure S2) and suggest that c.A453G is associated with pronounced and specific changes in predicted mutant mRNA structure, which are reflected in a large differences in calculated free energies between wild type and mutant mRNA.

### ALDH1A2 variants as possible modulators of the risk to CHD

#### Linkage disequilibrium and haplotype structure at the ALDH1A2 locus

**Figure 4:**
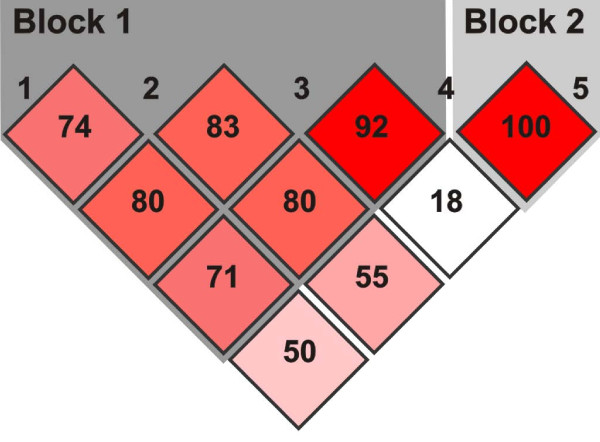
**Linkage disequilibrium blocks**. The structure of linkage disequilibrium between the single nucleotide polymorphisms shows two different blocks. The first one comprises markers 1 to 4 and the second block is formed by markers 5.

To investigate the hypothesis that variation at the ALDH1A2 locus modulates the risk of CHD we developed association studies based on polymorphic markers publicly available, including the c.A453G transition associated with spina bifida [24]. Polymorphic markers rs2704188; rs1441815; rs3784259; rs1530293 and rs1899430; were first utilized to determine the linkage disequilibrium structure (LD) and haplotype frequencies at the ALDH1A2 locus. Figure 4 indicates that the ALDH1A2 locus can be represented by two LD blocks. The first and largest LD block includes rs2704188; (13 kb upstream of translation start site), rs1441815 (10 kb downstream of exon 7) rs1530293 (37 kb downstream of the stop codon,), as well as rs3784259 (75 bp downstream of exon 8). Therefore, this large block includes sequences from the 5' region up to the 3' region. The second and smaller LD block contains the polymorphic marker rs1899430, which is located in the 3' untranslated region (39 kb downstream from the stop codon).

#### Genotype and Haplotype Association Analysis

The assayed markers, based on LD, frequency and distribution along the ALDH1A2 gene, were used in family-based association tests performed in 101 CHD trios. As displayed in Supplemental Table S4 (see additional file 1), none of the assayed marker genotype displayed any significant association with CHD when tested under TDT, or when tested under a standard family-based association test using parent genotypes as controls (not shown). Likewise, TDT analysis using CHD trios did not uncover ALDH1A2 haplotypes associated with significantly higher or lower risks of CHD in our population sample (see additional file 1 - Supplemental Table S5).

#### Association studies with the Ala151Ala silent mutation (c.A453G transition)

Because of the low population frequency of the c.A453G transition (and the anticipated low statistical power of TDT designs for this particular marker) we developed a case-control association study. To evaluate the frequency of the c.A453G transition, we sequenced 201 patients with TOF and a control group composed of 44 healthy individuals. c.A453G heterozygosity was present in 7 patients (3.5% of the CHD individuals), while in the control group 3 people were heterozygous for the variant (6,8% of healthy individuals). There was no statistical difference between genotype frequencies for this particular genetic marker.

## Discussion

In this study we set out to investigate if the ALDH1A2 gene is involved in human CHD. Our results indicate that there are, indeed, rare non-conservative mutations at deeply conserved positions in the ALDH1A2 molecule that carry an elevated risk of functional consequences. The frequency of these ALDH1A2 mutants is low, but not appreciably different from the frequency displayed by cardiac-enriched transcription factors such as Nkx2-5 or GATA-4 in sporadic CHD [39-41]. Therefore, our results with ALDH1A2 mutations in TOF are consistent with the emerging view that CHD is a highly heterogeneous disease that does not display a clear group of major genes responsible for most cases.

### Evidence for a functional importance of ALDH1A2 mutations in TOF

Several lines of evidence point towards the functional consequences of the observed mutations. First, they are very rare mutations, not present in any of the chromosomes from 150 normal individuals and only observed in two single probands from more than 100 screened CHD probands. Second, they lie in extremely conserved regions of the protein, as shown in Figure 1. Third, structural modeling of the mutated amino acids predicts severe shifts in ALDH1A2 spatial conformation, with disruptive influences in dimerization and tetramerization. The modeling strategy used in the present manuscript is robust. Molecular mechanics simulation has advanced to the point that it is possible to model mutations accurately, providing direct structural tests of enzyme catalysis models [42]. Moreover, ALDH1 enzymes are extremely well characterized from structural and functional standpoints [36,43-45] and their substrate preferences have been accurately established [44]. This makes this class of enzymes appropriate for structural modeling approaches that extract insights into enzyme properties without the necessity for biochemical analyses. Finally, the phenotypes associated with each of the two mutations that we describe are tightly linked to mature structures that receive critical contributions from embryonic tissues such as conotruncal ridges, atrioventricular cushions and muscular interventricular septum, tissues which have been previously documented as major targets for imbalances produced by genetic manipulation of the RA signaling pathway [10,46].

### Limited penetrance of ALDH1A2 mutations in TOF

The mutations that we described are present in unaffected parents of the studied probands. The simplest explanation for the lack of correlation between mutation and a cardiac phenotype in the parents of probands is that the mutation is not causally related to the phenotype. However, there are multiple and well known alternative reasons why mutations may not be associated with obvious cardiac manifestations in parents. In addition to producing exuberant cardiac phenotypes such as hypoplastic ventricles, large ventricular septal defects and double outlet right ventricle, mice haploinsufficient for the key retinoic acid receptor RXRalpha also display minor cardiac defects such as cleft mitral or tricuspid valve, disorganized trabeculae and displastic papillary muscles [10]. These subtle phenotypes may easily escape detection, either because they were manifested only transiently during embryogenesis, or because they are not enough to promote symptoms, even if present in adults. It is also well known that defective RA signaling stemming from Aldh1a2 haploinsufficiency in mice can be expressed as non-cardiac alterations such as vascular, laryngeal, tracheal, thymus and parathyroid defects [46]. Some of these non-cardiac defects are subtle and will only be detected by specific diagnostic procedures. In addition, they may not have reached enough physiological significance to be noticed at the time of physical examination of parents. Thus, extra cardiac defects may have gone unnoticed in parents and may not have been deemed important in the familiar history. Unfortunately, we were not able to screen other members of these families, nor could we submit the non-penetrant individuals, namely the father of the proband harboring the Ala151Ser mutation, nor the mother of the proband harboring the p.Ile157Thr mutation, to more detailed phenotypic assessments such as trans-esophageal echocardiography, or multislice computed tomography, which could have unveiled even some of the subtle phenotypes mentioned above. In sum, the ALDH1A2 mutations that we describe in TOF may not be fully penetrant. However, non-penetrance is not restricted to the mutations we described, but is, rather, a common feature in most, if not all, of the described genes that may cause CHD in humans such as PTPN11 in Noonan Syndrome [47], KCNQ1 in Long QT Syndrome [48] and troponin T in Familial Hypertrophic Cardiomyopathy [49].

Taken together, our data suggest that these rare mutations display a concrete potential to disrupt activity of the ALDH1A2 gene and contribute to the expression of congenital defects in CHD. Nonetheless, we acknowledge the tentative nature of our conclusions and point out that further functional assays and reproduction of these mutations in the context of genetically modified animals (e.g. knock-in mice) is required before any definitive conclusions can be reached.

### Association between the ALDH1A2 Ile157Thr mutation, TOF and Wilms Tumor

The complex phenotype displayed by the proband harboring the ALDH1A2 p.Ile157Thr mutation calls into question the possibility that TOF in this patient was caused by the same factor (s) underlying Wilms tumor disease. We believe this possibility is extremely unlikely. CHD and, in particular, TOF, is an exceptionally rare component in the constellation of Wilms tumor manifestations [50], with only two cases of TOF associated with Wilms tumor, but in the context of trisomy 18 [51,52]. Moreover, the vast majority of Wilms tumor cases are due to somatic mutations and only a minority is due to germ line mutations. Of germ line mutations, most are de novo cases and only a minority of those is manifested in families [53]. Furthermore, neither TOF, nor other cardiac outflow tract malformations have been reported for WT-1-null mice [54]. It is highly unlikely that the unilateral Wilms tumor displayed by the proband harboring the Ile157Thr is causally related to his TOF phenotype. We believe that the most probable scenario is that a germ line Aldh1a2 mutation is causally linked to TOF, while somatic mutation (s) are responsible for the unilateral Wilms tumor. Finally, it should be noted that no other syndromic features were observed in this patient, and that he did not present a clinical diagnosis compatible with other conditions associated with childhood tumors and congenital heart disease (i.e., Beckwith-Wiedemann Syndrome or the conditions associated with RAS/MAP kinase pathway mutations).

### Exon 4 as a hot spot for ALDH1A2 variants?

Our attention has been drawn by the fact that three out of five exonic alterations that we found in the ALDH1A2 gene in CHD are concentrated in exon 4, which encodes part of the tetramerization domain. The silent p.Ala151Ala mutation (c.A453G transition) and the non-conservative p.Ala151Ser mutation map to the same cistron, while the p.Ile157Thr is located only 6 cistrons apart. As demonstrated in Figure 3, the p.Ala151Ser impacts exon 4 splicing. p.Ala151Ser has a strong positive effect upon exon 4 splicing, virtually eliminating mRNAs that exclude exon 4, while p.Ala151Ala does not significantly decreases the ability to correctly splice exon 4. These results suggest that exon 4 is normally controlled by weak splicing signals and that it may be susceptible to splicing mutations. The skipping of exon 4 has severe consequences for the protein. Besides losing 60% of the tetramerization domain (23 amino acids), it introduces a frame shift after Ala121, which creates 26 aberrant amino acids followed by a termination codon that truncates the protein (data not shown). In summary splicing alterations involving exon 4 and the tetramerization domain are bound to have significant effects on protein structure and function.

### Lack of association between the silent mutation p.Ala151Ala (rs16939660) and CHD

Silent mutations have been reported to be associated and/or to produce phenotypes through multiple mechanisms such as: splicing effects, i.e. by disrupting splicing signals (reviewed in [55], or by changing mRNA folding/structure so as to induce conformational changes in the regions of exonic splicing enhancers that interfere with splicing factor binding (reviewed in [56]; mRNA stability [57,58], or due to the switch from common to rare codons, which could, in principle, produce delays in protein synthesis and changes in protein folding [59]. Therefore, the identification of a silent p.Ala151Ala mutation previously associated to spina bifida [24] in our group of TOF patients prompted us to investigate the potential functional consequences of this mutation in splicing and in predicted mRNA structure. We have shown that p.Ala151Ala is associated with a minor, non-significant reduction of exon 4 splicing and with a major predicted change in mRNA folding. Overall, we believe that those features of the p.Ala151Ala silent mutation merit further investigation, but that current evidence is not consistent with any sizeable functional effects in the specific context of CHD. Our current interpretation is supported by our study with the p.Ala151Ala mutation, which failed to provide evidence for an association between this polymorphism and CHD.

### Lack of evidence for ALDH1A2 as a modifier in CHD

In this work we also explored the possibility that the ALDH1A2 gene is a modifier of the risk to develop CHD. Using known polymorphic markers, we established that the ALDH1A2 gene encompasses only two LD blocks in the studied population and that none of the alleles investigated, whether, singly or in combination, were associated with a statistically significant protection or predisposition towards CHD. It should the highlighted, however, that the tested sample size would only have significant statistical power to detect effect sizes (odds ratio) in the order of 2,0 to 3,0, depending on the tested allele or haplotype (see Methods section).

## Conclusion

Our screen indicates that non-conservative ALDH1A2 mutations are associated with rare cases of human CHD and that ALDH1A2 polymorphisms (e.g. c.A453G) display functional differences in mRNA splicing and mRNA half-life that merit further functional investigation in the context of cell culture or genetically modified animals. In conclusion, our screen is the first pass of a research program to probe the role of ALDH1A2 in human CHD. While it represents a limited view of the CHD universe, it is sufficient to cast doubts on any major role for the ALDH1A2 gene as a determinant of CHD or as a modulator of its risk.

## Competing interests

The authors declare that they have no competing interests.

## Authors' contributions

Pavan, M.; Ruiz V.F.; Silva, F.A., Cravo R.M.; Vasconcelos, M. and Marques, L.P. conducted mutation screening, and genotyping; Sobreira, T.J.P. conducted structural modeling dynamics; Pavan, M. performed splicing experiments; Mesquita, S.M.F.; and Lopes, A.A.B. were responsible for patient selection; Oliveira, P.S.L. supervised structural modeling dynamics; Krieger, J.E.; Pereira, A.C., and Xavier-Neto J. conceived the investigation program. Pereira, A.C., and Xavier-Neto J designed the experiments and supervised the project. Pereira, A.C., Krieger, J.E. and Xavier-Neto, J. are responsible for the final draft of the manuscript. All authors read and approved the final manuscript.

## Pre-publication history

The pre-publication history for this paper can be accessed here:

http://www.biomedcentral.com/1471-2350/10/113/prepub

## Supplementary Material

Additional file 1 **Supplemental Tables**. Supplemental Table S1 to S5. Click here for file

Additional file 2 **Supplemental Figures legends**. Supplemental Figure S1 and S2 legends. Click here for file

Additional file 3 **Supplemental Figure S1**. A plot of the potential energy (in Kcal/mol) and RMSD (in angstroms) as a function of time for conformations obtained in a 6 ns molecular dynamics simulation. Click here for file

Additional file 4 **Supplemental Figure S2**. Impact of c.A453G variation in RNA structure. Click here for file
