# Supplementary material for: ALDH1A2 (RALDH2) genetic variation in human congenital heart disease
Source: BMC Med Genet. 2009 Nov 3;10:113. doi: 10.1186/1471-2350-10-113 (PMC2779186; doi:10.1186/1471-2350-10-113)
Supplement: Additional file 1 — Supplemental Tables. Supplemental Table S1 to S5. [file 1471-2350-10-113-S1.doc]

Supplemental Table 1: Primers used in ALDH1A2 gene sequencing.

| **ALDH1A2 Exons** | **Forward Primer** | **Reverse Primer** | **Fragment Length** |
| --- | --- | --- | --- |
| 1 | gggcgtcctaggtaggaa | cctcacccgctgaagaga | 371 bp |
| 2 | ttcctaaattggcgttgagtc | tgctttgtctatatctgcctgtta | 448 bp |
| 3 | tcagttgtgcctcttcctc | tttcagcagaatggcaaat | 364 bp |
| 4 | gatagtggttactggaagca | gatgtattaagtggcatgcc | 589 bp |
| 5 | actttctgggagtgacca | aacacacatcgctgagga | 186 bp |
| 6 | gagatttccaaggtcagtgctatt | aggaggaaacgtggctgatgaaag | 433 bp |
| 7 | cttcgggagtgcttctga | cctttgtgggcttgggta | 353 bp |
| 8 | taccccactaacttacatcttg | agtgctctttccccattatt | 435 bp |
| 9 | ctagggctttggagagg | aagggacagagaagcataagaaat | 462 bp |
| 10 | aaaaaccctaaaatgaaag | ttactgttgcaaaatgaata | 381 bp |
| 11 | gactcttcttgcataatctt | ggagtgtaccccttttct | 462 bp |
| 12 | tacactccttactgcttagat | gctccacgaaatgtttgttgaat | 367 bp |
| 13 | cgcatacgtgaggaatttttacat | ggtggccccttacagagtgc | 418 bp |

Supplemental Table 2: Markers and primer sequences used in the association study.

| **SNP** | **Forward Primer** | **Reverse Primer** | **Localization** | **Fragment Length** | **Alleles** |
| --- | --- | --- | --- | --- | --- |
| rs2704188 | tcccctaggctcctatctatgttatggatc | tccctttaagttgcccacag | 5’ region | 97 bp | C/T |
| rs1441815 | attaatgccattataaaagggcttgagaat | ggtgaacgacttcct | intron 7 | 113 bp | G/T |
| rs3784259 | gaaagtcatgcgagcaa | acatgaaccaccacaca | intron 8 | 426 bp | A/G |
| rs1530293 | cctccagtgtcccagagcccagacagtggt | gacgtggagagaggaagcag | 3’ region | 137 bp | A/G |
| rs1899430 | gctctagacctaaaaagccctagctttgaa | aaaatcatggaggaggagca | 3’ region | 179 bp | C/T |

Supplemental Table 3: 83 patients used in DHPLC study.

| **Patients** | **Disease** |
| --- | --- |
| 33 | Tetralogy of Fallot |
| 27 | Ebstein’s anomaly |
| 9 | Atrial Septal Defect |
| 6 | Ventricular Septal Defect |
| 1 | D-Transposition of the Great Arteries |
| 1 | Dextrocardia |
| 1 | Pulmonary Stenosis |
| 1 | Atrial-Ventricular Septal Defect |
| 1 | Truncus Arteriosus |

Supplemental Table 4: Allele Association Analysis.

| **SNP** | **Localization** | **Alleles** | **T : U** | **Chi Square** | **p Value** |
| --- | --- | --- | --- | --- | --- |
| rs2704188 | 5’ region | C/T | 42 : 38 | 0.20 | 0.07 |
| rs1441815 | intron 7 | G/T | 43 : 36 | 0.62 | 0.43 |
| rs3784259 | intron 8 | A/G | 44 : 43 | 0.01 | 0.91 |
| rs1530293 | 3’ region | A/G | 36 : 33 | 0.13 | 0.72 |
| rs1899430 | 3’ region | C/T | 29 : 28 | 0.02 | 0.89 |

Supplemental Table 5: Haplotype association analysis. No difference was verified by using a haplotype block of 4 or 5 SNPs.

| Block | **Haplotype Frequency** | **T : U** | **Chi Square** | **P value** |
| --- | --- | --- | --- | --- |
| CTAGC | 0.31 | 37.3 : 39.6 | 0.07 | 0.79 |
| TGGAC | 0.25 | 35.8 : 29.1 | 0.69 | 0.40 |
| TGAGC | 0.11 | 13.4 : 19.4 | 1.11 | 0.29 |
| TGGGT | 0.08 | 12.1 : 15.3 | 0.38 | 0.54 |
| TTAGC | 0.05 | 10.5 : 10.4 | 0.01 | 0.98 |
