# Supplementary material for: ALDH1A2 (RALDH2) genetic variation in human congenital heart disease
Source: BMC Med Genet. 2009 Nov 3;10:113. doi: 10.1186/1471-2350-10-113 (PMC2779186; doi:10.1186/1471-2350-10-113)
Supplement: Additional file 2 — Supplemental Figures legends. Supplemental Figure S1 and S2 legends. [file 1471-2350-10-113-S2.doc]

**SUPPLEMENTAL FIGURES LEGENDS**

**Supplemental Figure 1 -** **A plot of the potential energy (in Kcal/mol) and RMSD (in angstroms) as a function of time for conformations obtained in a 6ns molecular dynamics simulation.** RMSD values were calculated against the native conformation of ALDH1A2. The blue boxes represents the windows with the lowest energy (-8105 and -8296 Kcal/mol for p.Ala151Ser and Ile157Thr, respectively). The energy of the initial state from the native ALDH1A2 (-7936 Kcal/mol) is also shown (green dashed line).

**Supplemental Figure 2: Impact of c.A453G variation in RNA structure.** To establish how specific are the structural changes induced by the c.A453G variant in mRNA, we simulated the consequences of introducing other Alanine to Alanine silent mutations in the cistron GCA, which encodes Ala151. For that we introduced one transition in the first nucleotide (G to A) and another in the second nucleotide (C to T). As shown in supplemental figure 1, none of these simulated mutations produced any significant change in mRNA structure. To further contextualize the implications of the c.A453G we evaluated the changes induced by replicating the same A to G transition in all other GCA cistrons encoding alanine of the human ALDH1A2 gene. These controls were completed by modeling structural alterations induced by all other synonymous polymorphisms described at dbSNP (<http://www.ncbi.nlm.nih.gov/projects/SNP/>) for the ALDH1A2 gene. Supplemental figure 2 shows that only the c.A453G transition was associated with pronounced changes in predicted ALDH1A2 mRNA structure and free energy calculations.
