# Supplementary figures and images for: ALDH1A2 (RALDH2) genetic variation in human congenital heart disease
Source: BMC Med Genet. 2009 Nov 3;10:113. doi: 10.1186/1471-2350-10-113 (PMC2779186; doi:10.1186/1471-2350-10-113)

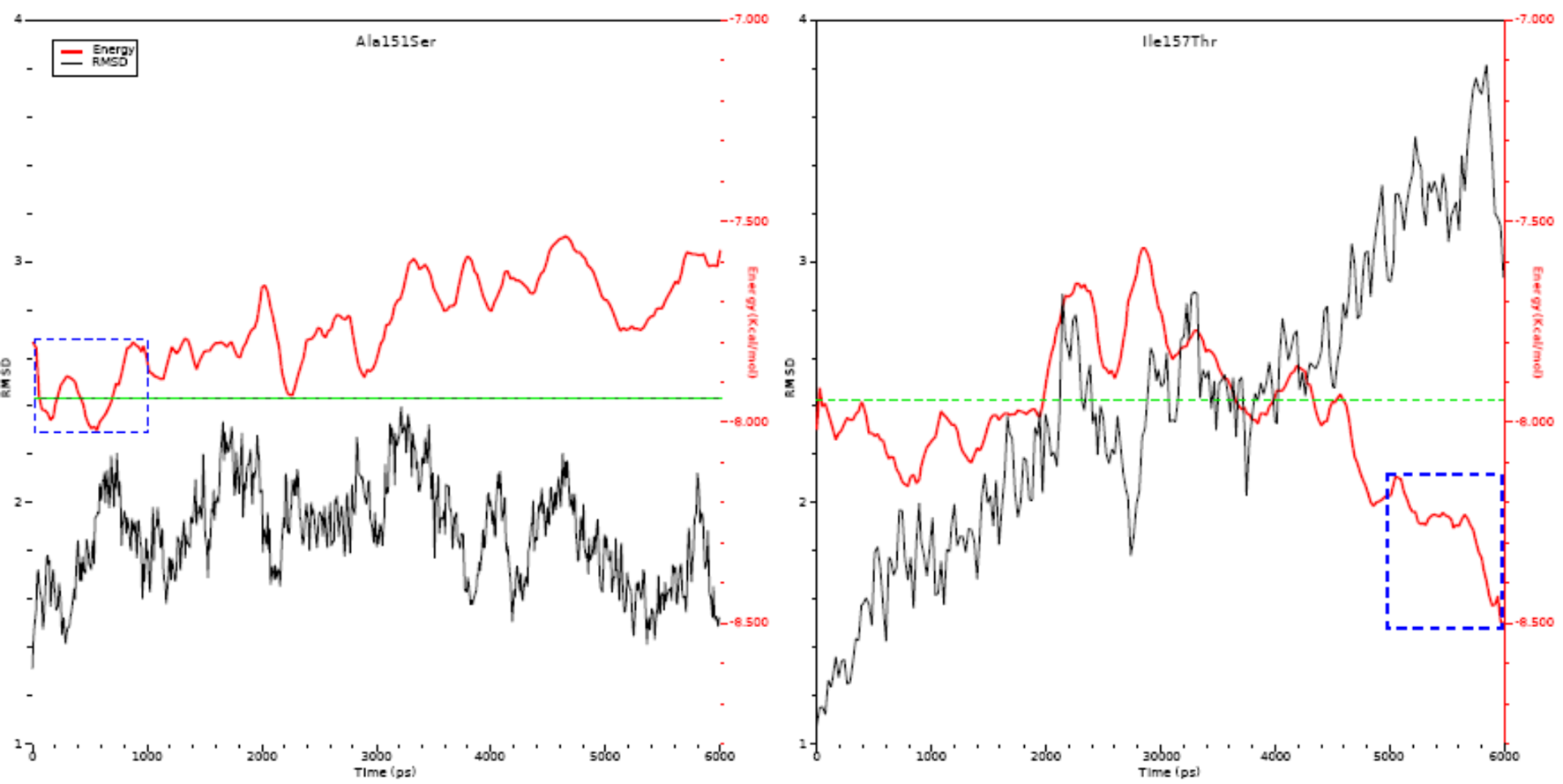

Supplement: Additional file 3 — Supplemental Figure S1. A plot of the potential energy (in Kcal/mol) and RMSD (in angstroms) as a function of time for conformations obtained in a 6 ns molecular dynamics simulation. [file 1471-2350-10-113-S3.tiff]

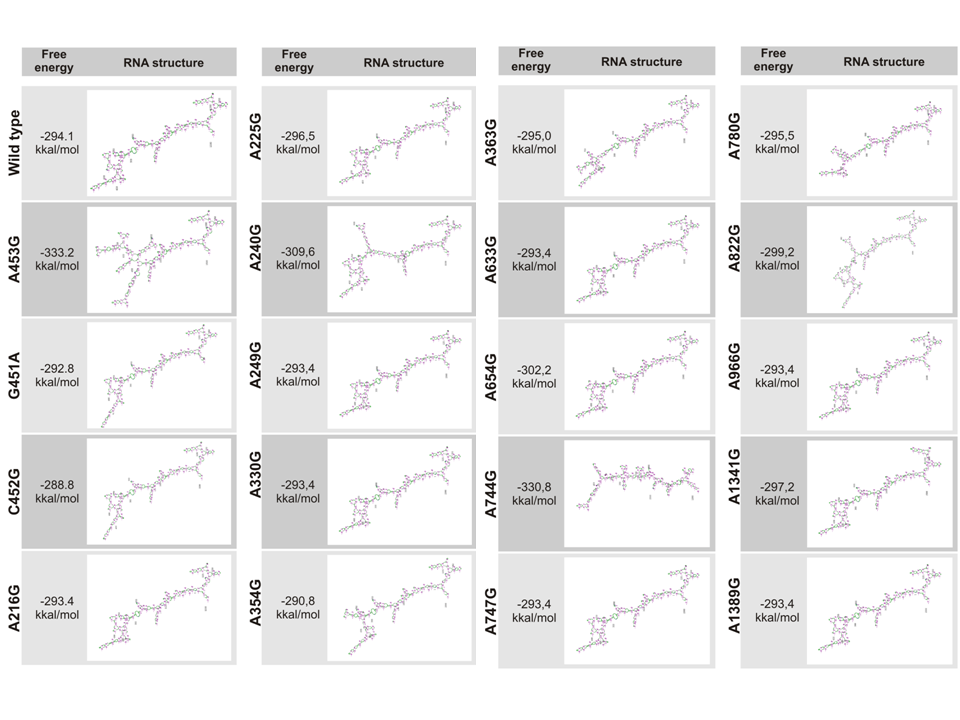

Supplement: Additional file 4 — Supplemental Figure S2. Impact of c.A453G variation in RNA structure. [file 1471-2350-10-113-S4.tiff]
